# Hyperglycemia-induced oxidative stress exacerbates mitochondrial apoptosis damage to cochlear stria vascularis pericytes via the ROS-mediated Bcl-2/CytC/AIF pathway

**DOI:** 10.1080/13510002.2024.2382943

**Published:** 2024-08-02

**Authors:** Tian-feng Shi, Zan Zhou, Wen-jun Jiang, Tian-lan Huang, Jun-qiang Si, Li Li

**Affiliations:** a Department of Physiology, Medical College of Jiaxing University, Jiaxing, Zhejiang, People's Republic of China; b Department of Physiology, Medical College of Shihezi University, Shihezi, Xinjiang, People's Republic of China; c The Key Laboratory of Xinjiang Endemic and Ethnic Diseases, Medical College of Shihezi University, Shihezi, Xinjiang, People's Republic of China; d Department of Physiology, Zhejiang Chinese Medical University, Hangzhou, Zhejiang, People's Republic of China

**Keywords:** High glucose, mitochondria, Ros, stria vascularis, pericytes, apoptosis, hearing loss, blood-labyrinth barrier

## Abstract

**Objectives:**

Diabetes is closely linked to hearing loss, yet the exact mechanisms remain unclear. Cochlear stria vascularis and pericytes (PCs) are crucial for hearing. This study investigates whether high glucose induces apoptosis in the cochlear stria vascularis and pericytes via elevated ROS levels due to oxidative stress, impacting hearing loss.

**Methods:**

We established a type II diabetes model in C57BL/6J mice and used auditory brainstem response (ABR), Evans blue staining, HE staining, immunohistochemistry, and immunofluorescence to observe changes in hearing, blood-labyrinth barrier (BLB) permeability, stria vascularis morphology, and apoptosis protein expression. Primary cultured stria vascularis pericytes were subjected to high glucose, and apoptosis levels were assessed using flow cytometry, Annexin V-FITC, Hoechst 33342 staining, Western blot, Mitosox, and JC-1 probes.

**Results:**

Diabetic mice showed decreased hearing thresholds, reduced stria vascularis density, increased oxidative stress, cell apoptosis, and decreased antioxidant levels. High glucose exposure increased apoptosis and ROS content in pericytes, while mitochondrial membrane potential decreased, with AIF and cytochrome C (CytC) released from mitochondria to the cytoplasm. Adding oxidative scavengers reduced AIF and CytC release, decreasing pericyte apoptosis.

**Discussion:**

Hyperglycemia may induce mitochondrial apoptosis of cochlear stria vascularis pericytes through oxidative stress.

## 1. Introduction

China is a major country with Diabetes mellitus (DM), and the prevalence rate of Diabetes has reached 11.2%, of which 90% are type 2 Diabetes patients [1]. Type 2 diabetes is a metabolic disease caused by insufficient insulin secretion or insulin resistance leading to elevated blood glucose [2]. If glycemia is not effectively controlled, the body will be in a high-glucose environment for a long period of time, leading to injury of the great vessels, micro vessels, nerves, etc., and eventually leading to a variety of serious complications, such as Diabetic neuropathy, Diabetic nephropathy, Diabetic retinopathy, etc. [3]. As research into diabetes has become more sophisticated, the hearing loss caused by diabetes has attracted increasing attention. In two reports presented at the Annual meeting of the American Diabetes Association (ADA) on June 7, 2009,it was pointed out that: High-frequency sensorineural hearing impairment can be a common but insufficient clinical diagnosis of diabetes complications [4], the data have shown that approximately 35% to 50% of individuals with diabetes have twice the incidence of hearing loss compared to nondiabetic individuals [5]. A number of researchers around the world have confirmed that there is a clear relationship between diabetes and hearing loss, but the specific pathogenesis remains unclear.

Microangiopaplasia is a major contributor to a variety of diabetic complications. High glucose levels in diabetic retinopathy will destroy the integrity of the blood-retinal barrier structure, leading to increased permeability and visual dysfunction [6–8]. The ear has a physical barrier similar to that of the blood-retina barrier: The blood-labyrinth barrier (BLB) of the cochlear stria vascularis, in the form of a highly differentiated capillary network in the ear, is located in the middle layer of the stria vascularis, and is a selective permeability physiological barrier between the blood and the labyrinth of the inner ear [9,10], which may maintain the homeostasis of the microenvironment of the inner ear. It has been reported that diabetes will also damage the structure of cochlear labyrinth barrier, resulting in increased permeability [11]. The barrier structure of cochlear labyrinth is mainly composed of endothelial cells (ECs), perivascular resident macrophage-like melanocytes (PVM/Ms) and pericytes (PCs) are composed [12]. The pericytes in the cochlear stria-blood labyrinth barrier, like the pericytes in the blood-retinal barrier, play an important role in angiogenesis, blood flow regulation and the integrity of the barrier [13,14]. In our previous research, we found that the reduction of cochlear pericytes can lead to an increase in the permeability of the blood-labyrinth barrier, thereby causing hearing impairment, suggesting that the of pericytes may represent a novel target for the prevention and treatment of diabetic hearing loss.

Chronic hyperglycemia induces an increase in reactive oxygen species (ROS), leading to cell apoptosis. Mitochondria are the central organelle for cellular aerobic respiration and also the hub for ROS production, cell apoptosis, and various signaling pathways [15]. Therefore, hyperglycemic oxidative stress is closely associated with mitochondrial-mediated cell apoptosis [16]. The question of whether diabetes induces apoptosis of cochlea pericytes through the mitochondrial pathway is one that requires further clarification. In our study, we utilized animal and cellular experiments to investigate whether hyperglycemia triggers pericyte apoptosis in the cochlear stria vascularis through the mitochondrial pathway, thereby resulting in hearing impairment, aiming to provide new insights into hearing loss caused by diabetes.

## 2. Materials and methods.

### 2.1. Animal grouping and treatment

Fifty 1-month-old 13–18 g male C57BL/6J mice were purchased from Beijing Weitong Lihua and started modeling after a one-week adaptation period in the breeding environment. Following a 4-week diet of high-fat food, the were fasted for 12 hours but had access to water. The day after the fast, they received an intraperitoneal injection of Streptozotocin (140 mg/kg). Seven days later, a fasting blood glucose measurement was conducted at 8 am and mice with blood glucose levels ≥11.1 mmol/L were classified as diabetic. The mice were housed in a room with a humidity of 40−50%, a temperature of 20–24°C, and a background noise level of 30 decibels. Diabetic mice with successful modeling were divided into control groups, a diabetic model 4-week group (DM4W), a diabetic model 8-week group (DM8W), and a diabetic model 12-week group (DM12W) based on the observation time. This study was approved the Medical Ethics Committee of the First Affiliated Hospital, School of Medicine, Shihezi University (A2020-167-01). All experiments were conducted in accordance with relevant guidelines and regulations. This study was carried out in compliance with the ARRIVE guidelines.

### 2.2. Auditory brainstem response (ABR)

The animals were placed in an acoustically shielded hearing testing room to maintain a background noise level of less than 30 dB(A). A heating pad set to 37°C was used to maintain a constant body temperature. The hearing threshold of different groups of mice was detected using a pa-800 (MADSEN, Denmark) device. Auditory brainstem response (ABR) testing employed three electrodes: the recording electrode was positioned at the center of the cranium, the reference electrode below the postauricular mastoid, and the ground electrode below the contralateral postauricular mastoid. The electrode impedance was maintained at less than 1 kΩ, and the stimulus sound headphones were inserted into the external auditory canal of the subject. The electrodes, ABR preamplifier, and sound stimulator used in the experiment were all placed in a dual acoustic and electrical shielded room. Other instruments and equipment were located outside the shielded area. An 8 kHz pure tone was selected, with each stimulus lasting 5 ms, a rise and fall time of 0.5 ms, a scanning time of 10 ms for each stimulus, and a filter bandwidth of 300–3000 Hz. An average of 1024 times was taken to obtain a reliable ABR waveform.

The sound stimulus was initially produced at 90 dB sound pressure level (SPL) and attenuated gradually by 10 dB as it approached the threshold. At the threshold level, the stimulus was attenuated an additional 5 dB SPL [17,18]. This method allowed for the identification of reproducible ABR waveforms, which were used to determine the ABR response threshold.

### 2.3. BLB permeability assessment

The permeability of the BLB was assessed by injecting 100 μl of 0.5% Evans Blue dye solution (G1810, Solarbio, China) via the tail vein. After complete circulation for 2 hours post-injection, both cochleae were excised. The cochae were fixed overnight at 4°C and rinsed with PBS three times the following day. The spiral ganglia of the cochlea were separated from the lamina terminalis and placed on glass slides, followed by observation under a fluorescence microscope (Olympus, Japan). Three random fields of equal area from each mouse's cochlear spiral ganglia were selected and analyzed for leakage using ImageJ.

### 2.4. Assessment of oxidative stress levels in various tissues

After deep anesthesia, 0.5 mL of venous blood from the inner canthus of each mouse was collected and placed in an EP tube. The blood was allowed to stand at room temperature for 30 min, then centrifuged at 300 r/min for 10 min to collect the supernatant. Subsequently, brain, liver, and cochlea tissues were harvested by heart perfusion, accurately weighed, and mixed with 9 times the volume of physiological saline in of weight (g): volume (mL) = 1:9. The mixture was homogenized on ice using a tissue grinder and centrifuged at 2500–3000 RPM for 10 min to remove the supernatant. Relative detection was performed according to the instructions the SOD (Jiancheng,Nanjing) and MDA (Jiancheng,Nanjing) kits.

### 2.5. Preparation of cochlear paraffin sections

After anesthetized with 2% pentobarbital sodium, the mice were perfused with PBS and 4% paraformaldehyde. After the neck was severed, both cochlea were removed quickly and placed in 4% paraformaldehyde for 24 h at 4°C. After decalcification with 10% EDTA for 7–10 days, the remaining bone of the specimen was removed and gradient dehydration was performed for transparency. The cochlea was immersed in wax in the direction of the parallel cochlea axis, and the cochlea was sectioned continuously (3 μm). Paraffin sections with intact tissue structure were selected for experiment.

### 2.6. HE staining

The cochlear paraffin sections were stained using a hematoxylin and eosin (HE) staining kit (C0105S, Beyotime, China). Hematoxylin staining was performed for 5 min, followed by washing for 3 s and eosin staining for 3 min. After washing with gradient ethanol and xylene to remove excess stain, the sections were coverslipped. Observation and photography were conducted under an optical microscope.

### 2.7. Immunohistochemistry staining

Immunohistochemistry was performed on cochlear paraffin sections from each group of mice to detect the expression of Caspase-3 and Bax. After antigen retrieval, the sections were washed three times with PBS (pH 7.4) for 5 min each time. The sections were then incubated with blocking reagent BSA for 30 min. The primary antibodies, anti-Caspase3 (1:200, Abcam, #ab32499) and anti-Bax (1:200, Abcam, #ab32503), were added and incubated overnight at 4°C. The sections were washed three times with PBS (pH 7.4) and incubated with secondary antibody, Goat Anti-Rabbit IgG H&L (1:1000, Abcam, #ab97051), for 2 hours. The sections were washed three times with PBS (pH 7.4) and then treated with DAB chromogen until the desired staining intensity was achieved. After washing in double-distilled water, the sections were stained with hematoxylin and mounted under a coverslip. The sections were observed under a microscope.

### 2.8. Immunofluorescence staining

The paraffin-embedded cochlear sections from different groups of mice were selected for immunofluorescence staining. After antigen retrieval, 0.3% Triton-100 solution was added to the sections for 30 min. Subsequently, the sections were blocked with 10% BSA solution at 37°C for 1 h. The sections were then washed with PBS three times for 5 min each. anti-Caspase-3(1:200, Abcam, #ab32499), anti-Bax (1:200, Abcam, #ab32503), and anti-PDGFR-β (1:200,Abcam,#ab69506) were applied to the sections and incubated overnight at 4°C in the dark. The next day, the slides were allowed to equilibrate to room temperature for 1 hour in a humid chamber, followed by three washes with PBS for 5 min each. Goat anti-mouse IgG H&L FITC(1:500,Abcam,ab6785) and Goat anti-rabbit IgG H&L TRITC (1:500,Abcam,#ab6718) secondary antibodies were applied to the sections and incubated in the dark for 60 min. The sections were then washed with PBS three times for 5 min each. After staining the nuclei with DAPI (1:1000) for 15 min, the sections were mounted with an anti-fluorescence quenching agent. Finally, imaging was performed using a fluorescence microscope.

### 2.9. Primary culture and identification of cochlear pericytes

At 7 days, 6–8 newborn C57BL/6J mices were anesthetized with 1% pentobarbitone sodium (40 mg/kg) and then sacrificed. Both cochlea were removed under aseptic conditions, the auditory vesicles were opened under microscope, the cochlea wall was exposed and the cochlea axis was taken out. Ophthalmic tweezers were used to tear off the entire stria vascularis and the spiral ligamentous tissue, and the stria vascularis tissue was carefully separated and cut into pieces. The cells were removed in a 3.5-cm dish with 700 μL of peripheral cell selection medium: an improved DMEM low-sugar culture medium (10% fetal bovine serum 0.1% Pigmented factor and 1% double resistance). Then the tissue blocks were placed flat and evenly in the petri dish and cultured in a constant temperature incubator at 37°C with 5% CO2 and 95% air. After 24 h, the unadhered cells and impurities were removed, and 1 ml of complete culture medium was carefully added. The color and appearance of the culture medium and the color of the tissue, as well as the cell growth, was observed daily. The pericytes could be purified by digestion and passage after 10–14 days.

Cells from generation P5 were selected for climbing, and the medium was discarded after the cells adhered to the wall on the second day. Briefly, cells were washed with PBS buffer for 3 times, 2 min/ time, and then fixed with 4% paraformaldehyde for a further 15 min. The cells were washed with PBS buffer for 3 times, 2 min/time, and the cells were permeated with Triton x-100 for 3 min, and washed with PBS for 3 times, 2 min/ time. After the closure solution was abandoned, pericyte specific markers anti-desmin (1:200,Abcam,#ab32362), PDGFR-β(1:200,Abcam,#ab69506)and endothelial cell marker vWF (1:200,Abcam, #ab287962) were added overnight at 4°C. The second day was rewarmed at room temperature for 30 min, washed with PBS buffer 3 times, 2 min/time, shielded from light and applied with secondary antibody at 37°C for 1 h, washed with PBS buffer 3 times, 2 min/time, and stained with DAPI for 15 min, washed with PBS buffer 3 times, 2 min/time, with anti-fluorescence quench agent, and observed under fluorescence microscope.

### 2.10. Cell Counting Kit-8 (CCK-8) screened the optimal concentration of high glucose treated pericytes

Using the 5th generation of primary cultured pericytes, after trypsin digestion for 3 min, the digestion was terminated with a serum-containing culture medium. The supernatant was discarded after centrifugation. The cells were mixed with 1 ml of culture medium and 10 microliters of cell suspension were added to the counting chamber. The pericytes were counted under a microscope, and the cell density was diluted to 5 × 10^3^ cells per well. The cell suspension was seeded in a 96-well plate for culture. The pericytes were intervened with different concentrations of glucose (5.5, 15, 25, 35, 45, 55 mmol/L) for 24 hours. After the intervention, the culture medium was discarded and the cells were washed with PBS. Then, 10 ml of CCK-8 working solution (Ape Bio,#K1018) was added to each well in the dark and incubated for 2 hours at 37°C. Finally, the changed 96-well plate was placed in an enzyme-labeled instrument and detected at a wavelength of 450 nm to measure the O.D. value. The cell activity of pericytes under different glucose concentrations was analyzed to determine the optimal high glucose intervention concentration.

### 2.11. Cell modeling and grouping

Cells were intervened with low glucose and high glucose culture mediums separately, with low glucose serving as the control group (NG). The high glucose group was treated with high glucose for 24 hours (HG24H), 48 hours (HG48H), and 72 hours (HG72H) after being starved in culture medium containing 1% serum for 12 hours. We designated the high glucose intervention group for 48 hours as the high glucose model group, and utilized N-acetylcysteine (NAC) for intervention. The groups at this time were the control group (NG), high glucose group (HG), high glucose + NAC group (HG + NAC), and NAC control group (NAC). In the quality control experiment assessing the performance of MitoSox, cells were divided into a high-glucose group (HG) and a high-glucose + Tiron group (HG + Tiron), where the glucose concentration in the HG group was consistent with that in other experiments, and Tiron concentration was 100 nM [19].

### 2.12. Flow cytometry with FITC-PI double staining

The cells in each group were digested with pancreatic enzyme for 3 min. The enzyme solution was aspirated and mixed with PBS to resuspend the cells. The cells were centrifuged at 1,000 r/min for 5 min. The supernatant was discarded, and 500 μl of 1× binding buffer was added to resuspend the cells. 5 μl of FITC and 10 μl of PI were added to mix evenly and react at room temperature in the dark for 10 min. Flow cytometry was performed within 1 hour.

### 2.13. Hoechst 33342 staining

Each group was treated with a 4 μmmol/L Hoechst33342 solution. After incubation for 15 min at 37°C, the cells were gently washed with PBS. Finally, 1 mL of PBS was added to each well for immediate observation under a fluorescence microscope. Bright blue cells indicated apoptotic cells. Image J software was used to calculate the apoptosis rate of cells in each well.

### 2.14. JC-1 and Mito-SOX combined with flow cytometry

Each group of cells was digested with trypsin for 3 min and observed under a microscope. At this time, the cells became round and were terminated with a 12% serum-containing culture medium as time went on. After gently blowing, the cells were transferred to a 4 mL EP tube and centrifuged at 1000 r/min for 5 min, and the supernatant was discarded. According to the instructions of the JC-1 kit (Solarbio, Beijing, China), the cells were suspended with a working solution of 10 μmol/L JC-1 and 5 μmol/L Mito-SOX, and cultured for 15 min in a 37°C incubator. After centrifuging at 1000 r/min for 5 min, the supernatant was discarded. The cells were suspended with 2 ml of PBS and centrifuged again at 1000 r/min for 5 min. The supernatant was discarded and 0.5 mL of PBS was used to suspend the cells for flow cytometry detection.

### 2.15. Extraction of cellular and mitochondrial proteins

After digestion with trypsin, cells were washed with PBS and collected by centrifugation at 1000 r/min for 5 min (approximately 2 × 10^7^ cells). The cells were then resuspended in 0.5 mL of ice-cold lysis buffer to allow cell recovery and transferred to a centrifuge tube. Subsequently, the cells were disrupted using an ultrasonic cell disruptor at 4°C and centrifuged at 1000 r/min for 5 min. The supernatant was collected and transferred to a new centrifuge tube, which was then centrifuged at 4°C and 1000r/min for 5 min. The supernatant was transferred to a new centrifuge tube and centrifuged at 12,000 r/min for 10 min at 4°C. The resulting supernatant contained cytoplasmic components and could be used for cytoplasmic protein extraction. The supernatant was transferred to a new centrifuge tube, and the mitochondria settled at the bottom of the tube. To the precipitate, 0.5 mL of Wash Buffer was added for resuspending the mitochondrial pellet, which was subsequently centrifuged at 1000 r/min for 5 min at 4°C. The supernatant was transferred to a new centrifuge tube and centrifuged at 12,000 r/min for 10 min at 4°C. The resulting supernatant was discarded, and the high-purity mitochondria were obtained as a pellet at the bottom of the tube. The pellet was resuspended in whole cell tissue lysis solution (180μ) and enzyme–substrate inhibitor (2μ), followed by ice-cold lysis for 20 min to obtain mitochondrial proteins.

### 2.16. Western blot

Cell or mitochondrial protein lysates were loaded onto a 10% Trisglycine polyacrylamide gel for electrophoretic separation. Thermo 26616 protein dual-color standard was used as the molecular weight marker. Subsequently, the proteins were transferred onto nitrocellulose membranes at 4°C, under 330 mA, using transfer buffer containing 20% methanol, 192 mM glycine, and 25 mM Tris for 2 hours. After washing with TBST, the membrane was incubated with blocking buffer (5% skim milk in TBST) for 1 hour, followed by overnight incubation at 4°C with primary antibodies against one of the following proteins: anti-Bcl-2 (1:1000, Abcam, #ab182858), anti-Bax (1:1000, Abcam, #ab32503), anti-Casepase-3 (1:1000, Abcam, #ab32499), anti-AIF (1:1000, CST, #5318), anti-cytochrome C (1:1000, CST, #4280), Anti-β-actin (1:10000, Invitrogen, #MA1-140) was used as the internal control for cellular protein, while anti-Cox (1:5000, Abcam, #ab202554) was used for mitochondrial protein. On the following day, after washing three times with TBST, the membrane was incubated with respective secondary antibodies, Goat anti-rabbit IgG (H + L) and Goat anti-mouse IgG (H + L) (Zhongshan Jinqiao, China), at room temperature for two hours. The membrane was then washed three times with TBST, followed by exposure and imaging using ECL chemiluminescent reagents on a imaging system for subsequent analysis.

### 2.17. Caspase 3 activity detection

According to the instructions of the Caspase 3 Activity Assay Kit (Beyotime, #C1115, China), each group of cells was treated. Briefly, proteins were extracted from each group of cells, the protein concentration in the samples was measured using the Bradford method. After normalization, 50 µL of the sample was mixed with 40 µL of buffer and 10 µL of substrate. Treated at 37°C in darkness for 4 hours. Then, the absorbance at 405 nm was measured using a microplate reader (Tecan).

### 2.18. Statistical analysis

Statistical analysis was performed using GraphPad Prism 9.5 software. Data are presented as the mean ± standard error (M ± SEM). Normality was assessed by Shapiro–Wilk or Kolmogorov–Smirnov tests. For normally distributed data, one-way ANOVA and Tukey's post hoc test were used for comparisons among multiple groups. For non-normally distributed data, the Kruskal–Wallis test and Dunn's multiple comparison were used. Details of sample size, replication, and specific statistical tests are provided in the figure legends. *P* values less than 0.05 were considered statistically significant.

## 3. Result

### 3.1. Diabetes results in permanent hearing loss and leakage of cochlear stria vascularis in C57BL/6J mice

**Figure 1.**
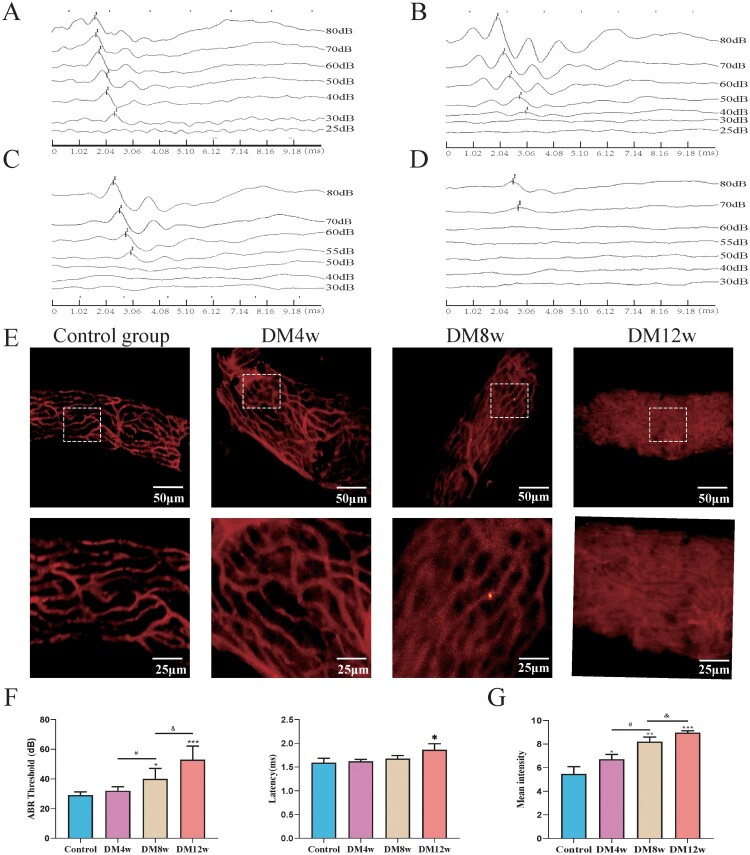
Diabetes results in permanent hearing loss and leakage of cochlear stria vascularis in C57BL/6J mice. A-D: Representative images of ABR waveforms in each group of mice (Control, DM4w, DM8w, DM12w); E: Representative images of Evans blue staining in each group of mice (Scale bar = 50 μm) and zoomed-in images (Scale bar = 25 μm); F: Statistical figure of ABR wave I hearing threshold of mice in each group and Latency statistics of ABR I wave, *n* = 10 mice per group, One-way Anova with Turkey’s post-hoc test, *P < 0.05, **P < 0.01,***P < 0.001 vs Control, #*P* < 0.05 vs DM4w group, &*P* < 0.05 vs DM8w group; G: Leakage fluorescence density statistical diagram of each group, *n* = 5 mice per group, One-way Anova with Turkey’s post-hoc test, *P < 0.05, **P < 0.01,***P < 0.001 vs Control, ^#^*P* < 0.05 vs DM4w group, ^&^*P* < 0.05 vs DM8w group. Data are presented as the means ± SEMs.

In Figure 1(A–D), we used auditory brainstem response (ABR) to assess the impact of diabetes on the hearing function of C57BL/6J mice. The ABR threshold and latency of I waves were determined. The ABR threshold was determined when the appearance of I waves preceded the last stimulation intensity or when I waves disappeared. As shown in Figure 1(F), the hearing thresholds of the DM8w and DM12w groups were significantly increased compared to the control group. The I wave latency in the DM12W group also showed a certain degree of increase. This suggests that diabetes impairs the hearing of mice. Furthermore, based on the results of the DM4w, DM8w, and DM12w groups, hearing deterioration further progresses with the duration of diabetes.

Concurrently, in Figure 1(E), the permeability of the organ of Corti was observed by intravenous injection of Evans blue dye into the mouse stria vascularis. Evans blue dye can bind to plasma albumin to label the cochlear stria vascularis, forming a red fluorescence. The permeability changes of the stria vascularis can be detected by observing the leakage of Evans blue within the cochlear stria vascularis. As shown in Figure 1(G), in the control group, red fluorescence was primarily observed within the microvessels, with minimal Evans blue leakage outside the microvessels. In the DM4, DM8w, and DM12w groups, however, red fluorescence appeared outside the microvessels, suggesting an increase in Evans blue leakage. The vascular outlines in the M12w group became almost invisible, indicating severe leakage in the stria vascularis of the cochlea. These groups exhibited a progressive increase in Evans blue leakage over time, with statistically significant differences noted.

### 3.2. Diabetes increases the oxidative stress levels in various organs of C57BL/6J mice and exacerbates with time

**Figure 2.**
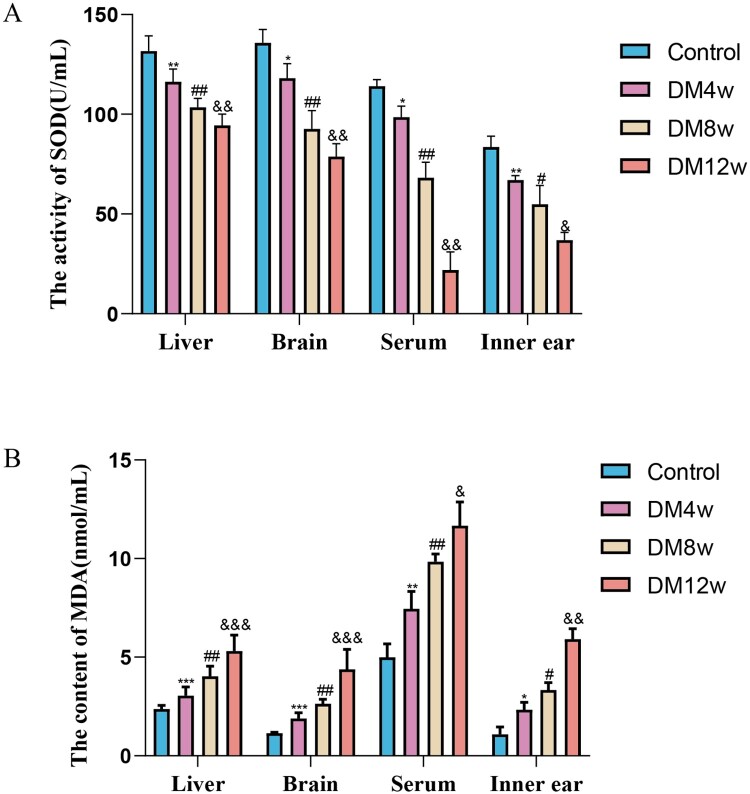
Diabetes elevates oxidative stress levels in mice's organs and exacerbates over time. A: Quantitative analysis of SOD expression levels in the liver, brain, serum, and cochlea of each group of mice, *n* = 5 mice per group, One-way Anova with Turkey’s post-hoc test, *P < 0.05, **P < 0.01 vs Control, #*P* < 0.05, ##*P* < 0.01 vs DM4w group, &*P* < 0.05, &&*P* < 0.01 vs DM8w group; B: Quantitative analysis of MDA expression levels in the liver, brain, serum, and cochlea of each group of mice, *n* = 5 mice per group, One-way Anova with Turkey’s post-hoc test, *P < 0.05, ***P < 0.001 vs Control, ^#^*P* < 0.05,^##^*P* < 0.01 vs DM4w group, ^&^*P* < 0.05,^&&^*P* < 0.01 and ^&&&^*P* < 0.001vs DM8w group. Data are presented as the means ± SEMs.

We used the TBA and WST-1 methods to determine the SOD and MDA contents in the liver, brain, serum, and cochlea of mice in each group. To clarify whether diabetes can lead to an increase in oxidative stress levels and the effects of different stages of diabetes on the oxidative stress levels of C57BL/6J mice. As illustrated in Figure 2, the SOD levels in various tissues of the diabetic model group mice were substantially lower than those in the control group. The MDA levels showed an increasing trend. This suggests that diabetes can elevate oxidative stress levels in mice, including the cochlea and other organs.

Concurrently, through the analysis of SOD and MDA expression levels in the organs of the DM4w, DM8w, and DM12w groups of mice, we found that with the prolonged duration of diabetes, the expression level of SOD in various organs continuously decreased. MDA expression levels continuously increased. This indicates that oxidative stress levels gradually increased with the progression of diabetes.

### 3.3. Diabetes induced cochlear stria vascularis injury and increased pericytes apoptosis in C57BL/6J mice

**Figure 3.**
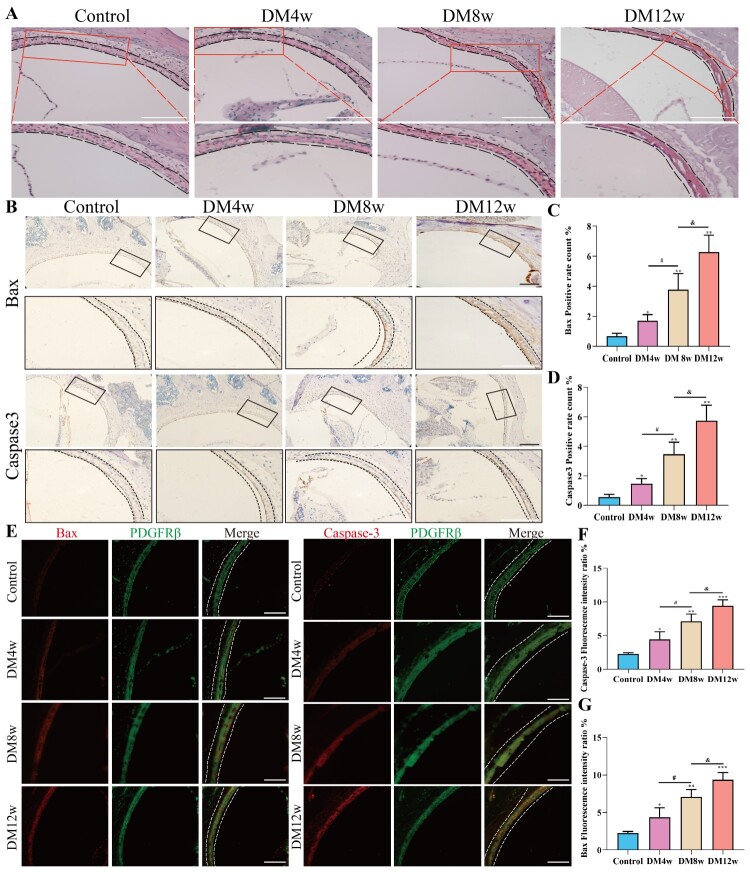
Diabetes induced cochlear stria vascularis injury and increased pericytes apoptosis in C57BL/6J mice. A: HE staining of cochlear stria vascularis in each group, Scale bar = 50 μm; B: B: Representative images of immunohistochemistry for Bax and Caspase-3 in the cochlear stria vascularis of each group, Scale bar = 50 μm; C: Statistical results of Bax expression levels in the cochlear stria vascularis, *n* = 5 mice per group, One-way Anova with Turkey’s post-hoc test, *P < 0.05, **P < 0.01 vs Control, ^#^*P* < 0.05 vs DM4w group, ^&^*P* < 0.05 vs DM8w group; D: Statistical results of Caspase-3 expression levels in the cochlear stria vascularis, *n* = 5 mice per group, One-way Anova with Turkey’s post-hoc test, *P < 0.05, **P < 0.01 vs Control, ^#^*P* < 0.05 vs DM4w group, ^&^*P* < 0.05 vs DM8w group; E: Representative images of co-labeling of PDGFR-β, Bax, and Caspase-3 in the cochlear stria vascularis pericytes, Scale bar = 50 μm; F: Statistical results of Bax expression levels in the cochlear stria vascularis pericytes, *n* = 5 mice per group, One-way Anova with Turkey’s post-hoc test, *P < 0.05, **P < 0.01, ***P < 0.01 vs Control, ^#^*P* < 0.05 vs DM4w group, ^&^*P* < 0.05 vs DM8w group; G:Statistical results of Caspase3 expression levels in the cochlear stria vascularis pericytes, *n* = 5 mice per group, One-way Anova with Turkey’s post-hoc test, *P < 0.05, **P < 0.01,***P < 0.001 vs Control, ^#^*P* < 0.05 vs DM4w group, ^&^*P* < 0.05 vs DM8w group. Data are presented as the means ± SEMs.

To further observe the effects of diabetes on the cochlear stria vascularis of C57BL/6J mice. We observed the morphological changes of the stria vascularis in each group of C57BL/6J mice through HE staining. As shown in Figure 3(A), the stria vascularis in the control group had normal morphology, clear structure, intact nuclear boundaries, and no rupture. In the DM4w group, the stria vascularis structure was relatively disordered, with a decrease in vacuoles and cytoplasm. DM8w showed a large number of vacuoles, nuclear condensation, and a reduction in cytoplasm. In DM12w, stria vascularis was more obvious, with stromal atrophy and an increase in vacuoles. In Figure 3(B), we detected the expression levels of Bax and Caspase-3 in the cochlear stria vascularis of each group of mice using immunohistochemistry. As shown in Figure 3(C,D), compared to the control group, diabetic mice showed increased expression levels of Bax and Caspase-3 in the cochlear stria vascularis. These expression levels increased gradually with the duration of diabetes, indicating that diabetes can elevate the apoptosis level of the cochlear stria vascularis.

We used the pericyte marker PDGFR-β to co-label with Bax and Caspase-3 to investigate the apoptosis level of pericytes in the cochlear stria vascularis. As shown in Figure 3(F,G), in the pericytes of the cochlear stria vascularis, the expression of Bax and Caspase-3 in diabetic mice increased, and this increase was gradual with the duration of diabetes. The trend was consistent with the expression levels of Bax and Caspase-3 in the cochlear stria vascularis. This indicates that diabetes may cause hearing loss by influencing the apoptosis of vascular pericytes.

### 3.4. Primary extraction and identification of cochlear stria vascularis pericytes and selection of high glucose intervention concentrations

**Figure 4.**
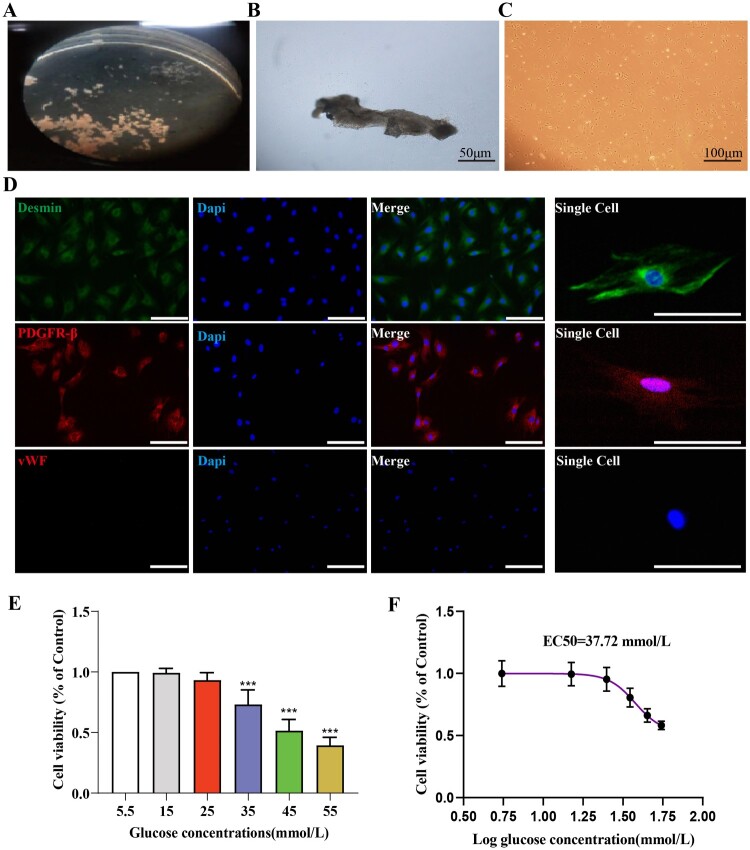
Successful primary culture of cochlear stria vascularis pericytes and optimal high glucose intervention concentration. A: Dissected cochlear stria vascularis; B: Representative image of stria vascularis pericytes on the third day of culture, Scale bar = 50 μm; C: Representative image of cochlear pericytes on the fourteenth day of culture, Scale bar = 100 μm; D: Identification of stria vascularis pericytes, Scale bar = 50 μm; E: Statistical results of the CCK8 assay for screening the appropriate high glucose intervention concentration, *n* = 6, One-way Anova with Turkey’s post-hoc test, ***P < 0.001 vs Control; F:The effect of glucose on the survival rate of stria vascularis pericytes and the EC50 of glucose for these cells.

Figure 4(A) illustrates the dissected stria vascularis. The stria vascularis was transferred to a special culture dish containing a special culture medium for adherent culture for 48 hours, then the medium was replaced. On the third day of cell culture, long spindle-shaped or irregularly shaped cells could be observed growing at the edge of the tissue, as shown in 4B. Approximately 10 days post-culture, PCs cells reached approximately 90% confluence, displaying a typical ‘paving stone’ arrangement, as illustrated in Figure 4(C). Around 14 days post-culture, the culture dish could be overgrown, and the first passage was performed, as shown in 4D. Primary cultured cells were labeled with the pericyte markers Desmin and PDGFR-β, showing positive Desmin and PDGFR-β expression and negative vWF expression, indicating the production of pure pericytes.

The glucose intervention concentration was screened using the CCK8 assay, and the results are shown in Figure 4(E). Cell viability decreased in a dose-dependent manner under different glucose concentrations. We also evaluated the EC50 of glucose for pericytes, and the results are shown in Figure 4(F), with the EC50 being 37.72 mmol/L. Based on these results, we chose 35 mmol/L as the hyperglycemic condition for subsequent experiments.

### 3.5. The apoptosis level of pericytes increases in a high-glucose environment

**Figure 5.**
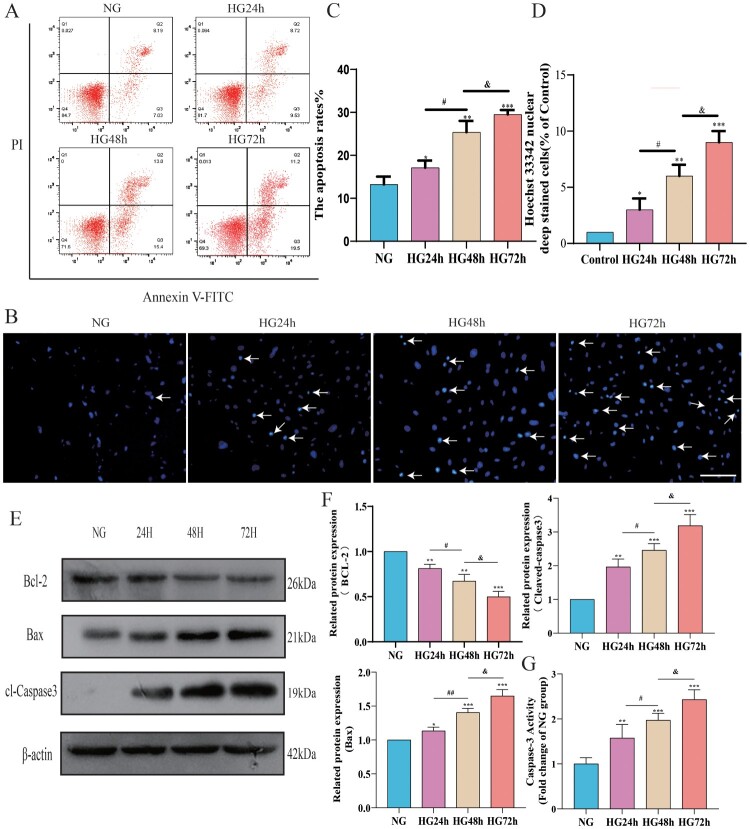
The apoptosis level of pericytes increases in a high-glucose environment. A: FITC-PI flow cytometry representative images; B: Hoechst33342 staining representative images (Hoechest 33342 deep stained cells have been marked with arrows), Scale bar = 50 μm; C: Statistical results of percentage of apoptotic cells by flow cytometry, *n* = 3, One-way Anova with Turkey's post-hoc test, *P < 0.05, **P < 0.01, ***P < 0.001 vs NG, ^#^*P* < 0.05 vs HG24 h, &*P* < 0.05 vs HG48 h; D: Statistical results of Hoechst33342 staining positive cells, *n* = 3, One-way Anova with Turkey's post-hoc test, *P < 0.05, **P < 0.01,***P < 0.001 vs NG, #*P* < 0.05 vs HG24 h, &*P* < 0.05 vs HG48 h; E: Western Blot representative images of Bcl-2, Bax and cl-Caspase3; F: Statistical results of expression levels of Bcl-2, Bax, Cleaved Caspase3 protein, *n* = 3, One-way Anova with Turkey's post-hoc test, *P < 0.05, **P < 0.01,***P < 0.001 vs NG,# *P* < 0.05,##*P* < 0.01 vs HG24 h, &*P* < 0.05 vs HG48 h; G. Statistical analysis of Caspase 3 activity results in each group of cells, *n* = 4, One-way Anova with Turkey's post-hoc test, **P < 0.01,***P < 0.001 vs NG,# *P* < 0.05 vs HG24 h, &*P* < 0.05 vs HG48 h. Data are presented as the means ± SEMs.

Figures 5(A,B) quantify the average apoptosis rate of pericytes in each group using flow cytometry and Hoechst33342 staining, respectively. Flow cytometry analysis results are shown in Figure 5(C), where the apoptosis rate in the HG24 h, HG48 h, and HG72 h groups significantly increased. Furthermore, the apoptosis rate of pericytes increased to a certain degree as the duration of high-glucose intervention extended. Figure 5(D) displays the Hoechst33342 staining results, indicating a significant rise in the proportion of Hoechst33342-positive cells following high-glucose treatment, and this proportion also increased with the prolonged high-glucose intervention.

To further explore the impact of a high-glucose environment on the apoptosis level of pericytes, we conducted a Western Blot experiment to detect the anti-apoptotic protein Bcl-2, the pro-apoptotic protein Bax, and cl-Caspase3. As shown in Figure 5(F), after high-glucose treatment, the expression level of Bcl-2 in pericytes decreased, while the expression levels of Bax and cl-Caspase3 increased, indicating that high glucose can induce pericyte apoptosis. By comparing the HG24 h group, HG48 h group, and HG72 h group, we found that the longer the duration of high-glucose treatment, the lower the Bcl-2 expression level and the higher the expression levels of Bax and cl-Caspase3, indicating a more severe apoptosis. As for the detection of Caspase 3 activity, the results are shown in Figure 5(G), where Caspase 3 activity increased with the duration of high glucose intervention. These findings are consistent with the results of the FITC-PI flow cytometry and Hoechst33342 staining, further confirming that in a high-glucose environment, pericyte apoptosis levels increase over time.

### 3.6. After high-glucose intervention the mitochondrial ROS content of pericytes increases in a time-dependent manner

**Figure 6.**
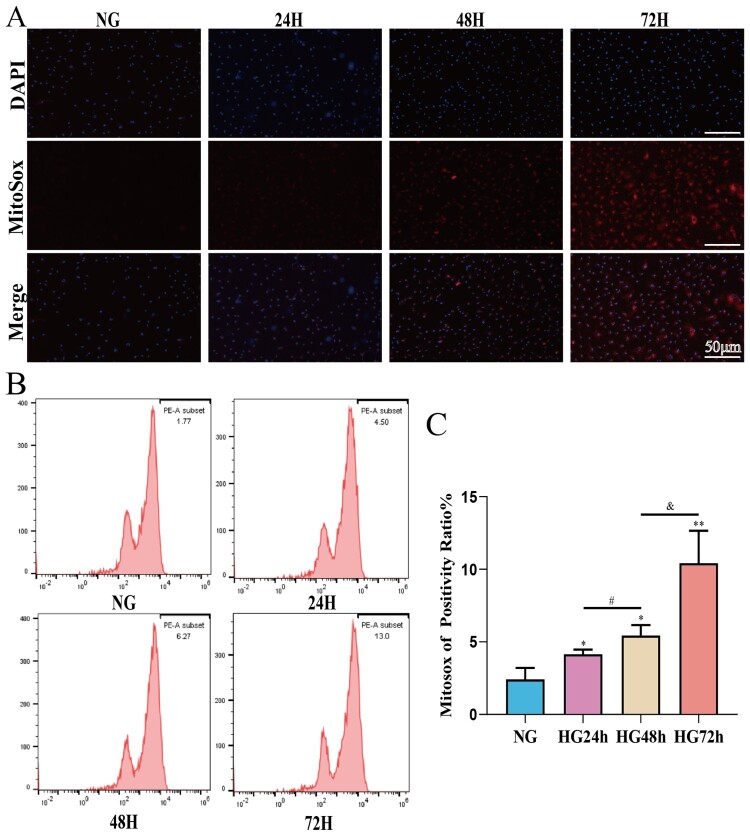
The mitochondrial ROS content of pericytes increases in a time-dependent manner in a high-glucose environment. A: Representative fluorescent image of Mitosox, Scale bar = 50 μm; B: Representative flow cytometry image combining Mitosox; C: Statistical analysis of mitochondrial ROS content, *n* = 3, One-way Anova with Turkey’s post-hoc test, *P < 0.05, **P < 0.01, ***P < 0.001 vs NG, ^#^*P* < 0.05 vs HG24 h, ^&^*P* < 0.05 vs HG48 h. Data are presented as the means ± SEMs.

To investigate the effect of high glucose on the mitochondrial ROS content in pericytes, Mitosox combined with flow cytometry was used to detect the ROS content in pericyte mitochondria. We used Tiron to intervene in pericytes cultured in high glucose to assess the monitoring effect of the Mitosox probe on mitochondrial ROS (Supplementary Figure S1). After confirming the monitoring role of MitoSox on mitochondrial ROS in pericytes, we measured the mitochondrial ROS levels in each group of cells. As shown in Figure 6(C), compared to the NG group, the mitochondrial ROS content in the pericytes of the HG24 h, HG48 h, and HG72 h groups significantly increased. Moreover, the mitochondrial ROS content increased in a time-dependent manner from HG24 h to HG48 h, and further to HG72 h. This suggests that high glucose may lead to a time-dependent increase in the mitochondrial ROS content of pericytes.

### 3.7. The reduction in mitochondrial membrane potential in pericytes induced by high glucose is time-dependent

**Figure 7.**
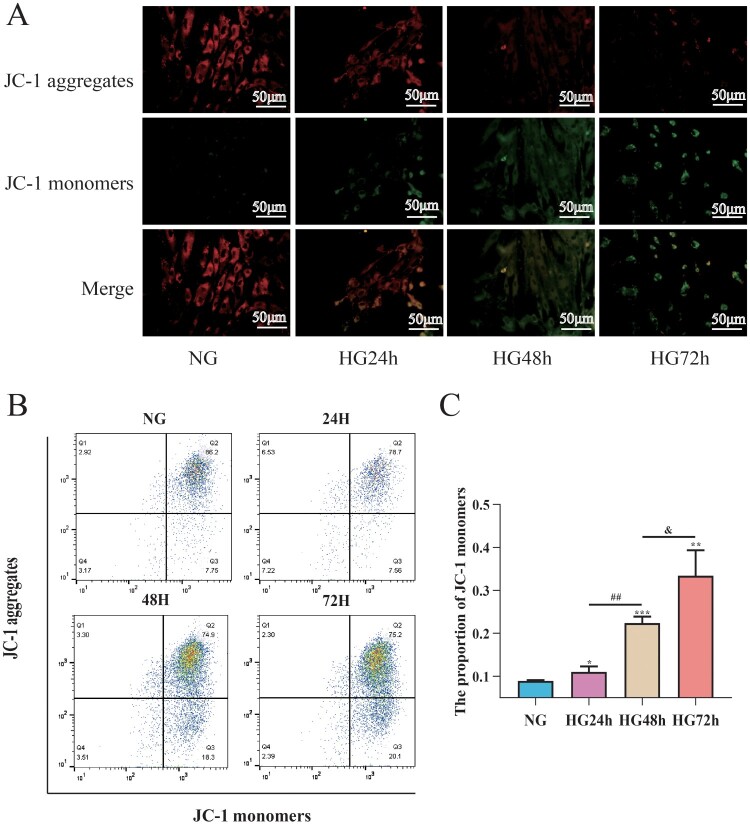
High glucose can lead to a decrease in mitochondrial membrane potential in pericytes and this decrease is time-dependent. A: Representative fluorescent image of JC-1, Scale bar = 50 μm; B: Representative flow cytometry image combining Mitosox; C: Statistical analysis of mitochondrial membrane potential, *n* = 3, One-way Anova with Turkey’s post-hoc test, *P < 0.05, **P < 0.01, ***P < 0.001 vs NG, ^##^*P* < 0.01 vs HG24 h, ^&^*P* < 0.05 vs HG48 h. Data are presented as the means ± SEMs.

To assess the effects of high glucose on mitochondrial membrane potential in pericytes, JC-1 combined with flow cytometry was used to detect mitochondrial membrane potential in pericytes. As shown in Figure 6(C), compared to the NG group, the mitochondrial membrane potential of pericytes in the HG24 h, HG48 h, and HG72 h groups significantly decreased; compared to the HG24 h group, the mitochondrial membrane potential in the HG48 h group decreased; compared to the HG48 h group, the mitochondrial membrane potential in the HG72 h group decreased, suggesting that high glucose can lead to a decrease in mitochondrial membrane potential in pericytes, which is time-dependent (Figure 7).

### 3.8. High glucose induces pericyte apoptosis through the mitochondrial pathway mediated by oxidative stress involving CytC/AIF

**Figure 8.**
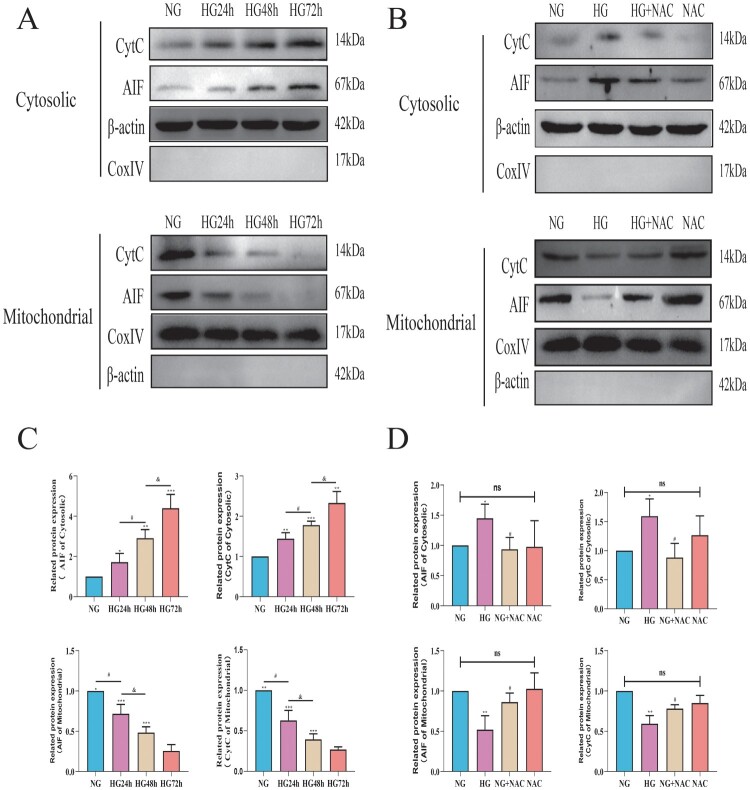
Oxidative stress induced by high glucose mediates the expression changes of CytC/AIF between the cytoplasm and mitochondria. A: Representative Western blot images of CytC and AIF in the cytoplasm and mitochondria of pericytes cultured under high glucose; B: Representative Western blot images of CytC and AIF in the cytoplasm and mitochondria of pericytes cultured under high glucose with NAC supplementation; C: Quantitative analysis of CytC and AIF expression in the cytoplasm and mitochondria of pericytes cultured under high glucose, n = 3, One-way Anova with Turkey’s post-hoc test, *P < 0.05, **P < 0.01, ***P < 0.001 vs NG, ^#^*P* < 0.05 vs HG24 h, ^&^*P* < 0.05 vs HG48 h; D: Quantitative analysis of CytC and AIF expression in the cytoplasm and mitochondria of pericytes cultured under high glucose with NAC supplementation, *n* = 3, One-way Anova with Turkey’s post-hoc test, *P < 0.05, **P < 0.01 vs NG, ^#^*P* < 0.05 vs HG. Data are presented as the means ± SEMs.

To investigate the effects of high glucose on the expression of AIF and CytC in pericyte mitochondria. We extracted cellular and mitochondrial proteins for Western blot analysis to detect the expression levels of AIF and CytC in both pericyte mitochondria and cytoplasm. As shown in Figure 8(C), with the prolonged duration of high glucose intervention, AIF and CytC were gradually released from mitochondria into the cytoplasm of pericytes, suggesting that high glucose can indeed induce pericyte apoptosis through the mitochondrial pathway. To further investigate whether this mitochondrial apoptosis induced by high glucose is mediated by ROS, we added the oxidative scavenger NAC to the high-glucose intervention. As shown in Figure 8(D), upon addition of NAC, the release of AIF and CytC from pericyte mitochondria into the cytoplasm was significantly reduced, suggesting that high glucose-induced oxidative stress mediates changes in the expression of CytC/AIF between the cytoplasm and mitochondria.

**Figure 9.**
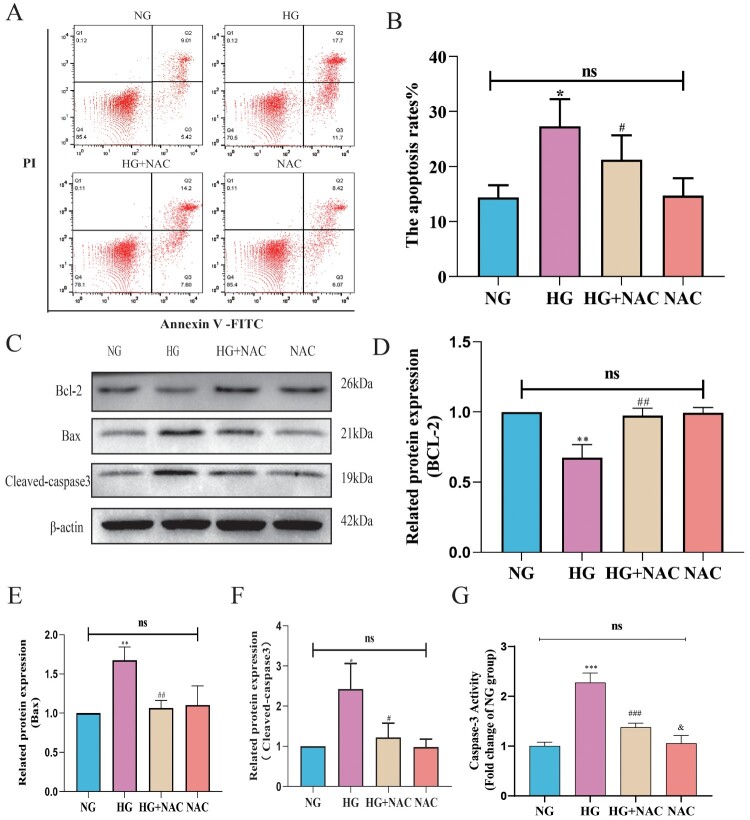
High glucose induces pericyte apoptosis through the mitochondrial pathway mediated by oxidative stress involving CytC/AIF. A:Representative images of FITC-PI flow cytometry; B: Statistical analysis of the percentage of apoptotic cells by flow cytometry, *n* = 3, One-way Anova with Turkey’s post-hoc test, *P < 0.05 vs NG, ^#^*P* < 0.05 vs HG; C: Representative Western Blot images of Bcl-2, Bax, and cleaved-Caspase3; D: Statistical analysis of Bcl-2 protein expression level, *n* = 3, One-way Anova with Turkey’s post-hoc test, **P < 0.01 vs NG, ^##^*P* < 0.01 vs HG; E: Statistical analysis of Bax protein expression level, *n* = 3, One-way Anova with Turkey’s post-hoc test, **P < 0.01 vs NG, ^##^*P* < 0.01 vs HG; F: Statistical analysis of cleaved-Caspase3 protein expression level, *n* = 3, One-way Anova with Turkey’s post-hoc test, *P < 0.05 vs NG, ^#^*P* < 0.05 vs HG, h; G. Statistical analysis of Caspase 3 activity results in each group of cells, *n* = 4, One-way Anova with Turkey's post-hoc test, ***P < 0.001 vs NG,### *P* < 0.05 vs HG, &*P* < 0.05 vs HG + NAC. Data are presented as the means ± SEMs.

Subsequently, we employed NAC to regulate the oxidative stress level induced by high glucose and utilized flow cytometry to detect the apoptosis level of pericytes following NAC intervention. As shown in 8B, compared to the HG group, the apoptosis rate of pericytes in the HG + NAC group was reduced. We further examined the expression levels of apoptotic proteins Bax, cleaved-caspase3, and anti-apoptotic protein Bcl-2 using Western blot analysis. As depicted in 8D-F, compared to the HG group, the expression of Bax and cleaved-caspase3 was decreased, while the expression of Bcl-2 was increased in pericytes of the HG + NAC group. The results of the Caspase 3 activity assay were consistent with the trend observed in the Western blot results, as shown in Figure 9(G). Compared to the HG group, the Caspase 3 activity in the HG + NAC group cells significantly decreased. These results suggest that high glucose induces pericyte apoptosis through the mitochondrial pathway mediated by oxidative stress involving CytC/AIF.

## 4. Discussion

Jordao [20] was the first to propose a link between diabetes and hearing loss. Since then, the association between diabetes and hearing loss has been a topic of research and debate [21–23]. The National Health and Nutrition Examination Survey found that high-frequency hearing loss occurs in over 70 percent of diabetics between the ages of 50 and 69. Previous studies have found that the incidence of hearing loss in diabetic patients is about twice that in non-diabetic patients, and it is closely related to the course of disease. The incidence of hearing loss in DM patients with a course of disease of more than 10 years is higher than that in DM patients with a course of disease of less than 10 years [24–26]. However, due to the invisibility of diabetic hearing loss, because of the slow course of the disease, diabetic hearing loss is often ignored by people, and due to the complex structure and deep location of the inner ear and the limitation of bone wrapping, there are relatively few studies on the mechanism of hearing loss caused by diabetes. Clarification of the pathogenesis of diabetic hearing loss is therefore of great importance for the early diagnosis, prevention, and treatment of diabetes [27–29]. In this study, we took C57BL/6J mice as the object of investigation to explore whether apoptosis of the capillary pericyte in the cochlear stria of C57BL/6J mice is mediated by the mitochondrial pathway in the high-glucose environment in diabetes. Thereby affecting the permeability of the blood labyrinth barrier. These data demonstrated that high glucose can mediate the apoptosis of capillary pericytes in the cochlear stria of C57BL/6J mice through the mitochondrial pathway, and affect the permeability of the blood labyrinth barrier, providing experimental basis for revealing the specific mechanism of diabetic hearing loss.

Because of diabetic patients in the study of easily influenced by many factors such as age, drugs, duration, noise interference, makes the study of diabetic hearing loss results are different, so this study by high-fat joint STZ build animal model of type 2 diabetes [30,31], to a certain extent, can reduce the interference of the above factors. In addition, histopathological studies can be conducted to further explore the possible mechanism of hearing impairment in diabetes [32]. It is a common method to construct an animal model of type 2 diabetes mellitus by combining high fat with STZ [33,34], which can better evaluate the drugs for the treatment of type 2 diabetes and conduct researches on the pathological mechanism and complications of type 2 diabetes, and has the advantages of simplicity, economy and time saving [35]. Auditory brainstem response ABR has good repeatability and stability, and can accurately reflect hearing level [36,37], which is one of the effective methods for clinical objective evaluation of early auditory center lesions in diabetes patients [38,39]. In this study, C57BL/6J mice were divided into normal group, diabetic 4W group, diabetic 8W group and diabetic 12W group, and the effect of diabetes on the auditory function of mice was observed by ABR threshold detection. In comparison to the normal group, the results showed that the hearing threshold for diabetes mellitus gradually increased with prolongation of the disease course in a time-dependent manner, further confirming that diabetes does indeed cause hearing impairment and is closely related to the course of the diabetes.

Diabetic microangiopathy is an important factor in diabetes cause hearing loss [40], microcirculation, especially involving blood vessels grain is the important pathologic basis of diabetic deafness blood vessels grain as main capillaries within the cochlea, in the cochlear lateral wall, external connected with spiral ligament, are connected to the endolymph inside, mainly by the edge of the cell [41]. Cochlear stria atrophy is a common pathological result of various inner ear diseases [42,43]. Numerous studies have found that thickening of the cochlear vascular wall and atrophy of the stria capillaries in diabetic mice [44,45]. As shown by HE staining results on paraffin tissue sections from the cochlea in the present experiment, compared with the normal group, the Stria Vascularis in the diabetic group was significantly atrophic, and the degree of atrophy progressively increased with extension of the course of the disease. Studies have shown that diabetes will damage the structure of the cochlear blood maze barrier, resulting in increased permeability [11]. As shown in the Evans Blue experiment of this study, compared with the normal group, Evans blue leakage of capillaries in the diabetic group increased, and red fluorescence could be seen in the space outside microvessels. Evans Blue leakage increases gradually.

Type 2 diabetes affects pericyte number, function, and phenotype in a wide range of tissues. These include pancreas, retina, kidney and other tissues [46,47]. PCs in the stria vascularis of cochlea, as the main component of the vascular labyrinth barrier, play an important role in maintaining the permeability of the barrier. Therefore, the present study took the pericyte vascularis of cochlea as the main object of study. To explore the possible mechanisms of diabetic hearing loss. Similarly, immunofluorescence and immunohistochemistry were performed on paraffin tissue sections of cochlea, and the results showed that with the prolongation of the course of diabetes, the expressions of apoptotic proteins Bax and Casapse3 in pericytes of cochlea gradually increased, suggesting that diabetes can induce the apoptosis of pericytes, and their functions and phenotypes will also change correspondingly. In order to further explore the specific mechanism of diabetes-induced apoptosis of cochlear pericytes, we conducted primary culture of cochlear pericytes, and then treated the primary cultured pericytes with high glucose for different periods of time, which were divided into NG group, HG24 h, HG48 h and HG72 h groups. Flow cytometry and Hochest33342 technique were used to detect the apoptosis rate of pericytes under high glucose intervention. The results were consistent with in vivo immunofluorescence and immunohistochemistry experiments. The apoptosis rate of pericytes increased with the prolonged time of high glucose intervention.

Apoptosis induced by mitochondrial injury is one of the classical apoptosis pathways. In recent years, studies have proposed that high glucose or high lipid in diabetes can lead to increased mitochondrial load and ROS [48], leading to a series of mitochondrial function and structure changes. Xing L [49] found that high glucose can increase mitochondrial ROS in renal podocyte and then induce podocyte to undergo mitochondrial pathway apoptosis, and Zhang Yuan [50] found the same situation in retinal pigment epithelial cells. However, it is not clear whether high glucose also mediates the apoptosis of cochlear pericytes through the mitochondrial ROS pathway.

Indeed, it is well known that mitochondria-induced apoptosis can be divided into caspase dependent and non-dependent pathways [51,52]. When high concentration of ROS is generated in mitochondria, mitochondrial damage can be caused [53–56]. And the damage of mitochondria outer membrane permeability increased, mitochondria swelling, inhibits PTP opening in activation, in turn, led to a decline in mitochondrial membrane potential and mitochondrial membrane potential drop can make the respiratory chain and oxidative phosphorylation coupling, mitochondrial matrix osmotic pressure increases, membrane swelling, mitochondrial intermembrane space of CytC, AIF released into the cytoplasm. CytC in the cytoplasm will form apoptotic bodies with APAF-1, which further activate Procaspase-9 and Procaspase-3, thereby activating the caspase apoptosis pathway. AIF accumulates in the cytoplasm and will eventually translocate to the nucleus, leading to breakage of nuclear DNA and thereby activation of the caspase-independent apoptosis pathway [57].

And the mitochondrial outer membrane permeability family BCL – 2 plays an important regulatory role, on the one hand, its apoptosis factors such as Bax may increase the permeability of the outer membrane of mitochondria, to further promote apoptosis mediated by mitochondrial damage, on the other hand, its apoptosis suppressor factor like BCL-2 may decrease the permeability of the mitochondrial outer membrane, in order to mitigate apoptosis mediated by mitochondrial damage [58].

To determine if the mitochondrial apoptosis pathway plays a role in diabetes induced apoptosis of cochlear pericytes, in the present study, we used Mitosox combined with flow cytometry and JC – 1 combined with flow cytometry at the cellular level to detect mitochondrial ROS content and mitochondrial membrane potential of pericytes in the presence of a high glucose intervention, and the results demonstrated that: with prolongation of the high glucose intervention period, a gradual increase in mitochondrial ROS content was observed in pericytes of the cochlea and the membrane potential also showed a time-dependent increase. In addition, this experiment on the basis of taking weeks cells mitochondria, using Western blot technique to observe high sugar intervention cells mitochondrial CytC and AIF next week to release the cytoplasm, the results show that compared with NG group, high sugar intervention cells mitochondrial CytC and AIF next week to cytoplasmic release, and time dependence. However, NAC can reduce the mitochondrial ROS content, reduce the potential of peripheral cell membrane, and alleviate the release of CytC and AIF from peripheral cell mitochondria to cytoplasm.

Meanwhile, NAC can also eliminate cisplatin-induced oxidative stress in pericytes of the cochlear stria vascularis, thereby reducing the ototoxicity of cisplatin. In our previous study, we also found that quercetin, a flavonoid compound widely found in nature, can reduce cisplatin-induced oxidative stress levels in pericytes of the cochlear stria vascularis and can act as a scavenger of oxidative stress in these cells [42]. In the other two barriers: the blood–brain barrier and the blood-retinal barrier, the elimination of oxidative stress in pericytes is a concern for researchers. In the blood-retinal barrier, studies have found that oxidative stress induced by high glucose originates from NADPH oxidase derivatives. Inhibition of NADPH oxidase by Apocynin can reduce pericyte apoptosis in retinal pathology [59,60]. The GLP-1 receptor may also play a crucial role in oxidative stress at the blood-retinal barrier. Using the GLP-1 receptor agonist Lixisenatide can alleviate oxidative stress. Lixisenatide controls the inflammatory response caused by oxidative stress by regulating the Est2 [61]. As reactive oxygen species (ROS) accumulate, the GPx activity and GSH levels in pericytes decrease [62], which may subsequently result in ferroptosis. Intervention through iron chelators and the mechanisms of iron uptake in pericytes may also mitigate oxidative stress levels in pericytes [63].

Because the retina and brain contain microglia and other cells that are absent in the blood-labyrinth barrier, the pathways causing oxidative stress in pericytes may be more diverse. Studies have found that microglial activation can promote pericyte apoptosis via ROS [64]. Therefore, drugs that reduce microglial activation in these two regions may act as antioxidants to reduce pericyte damage. Numerous compounds have been investigated as scavengers of oxidative stress in pericytes within the blood–brain barrier, including Topiramate, Acetazolamide, and Mitochondrial carbonic anhydrase VA [65–67]. Current studies also focus on the molecular targets of oxidative stress in pericytes, aiming to reduce oxidative damage by regulating the relevant molecular pathways. For instance, in oxidative stress in pericytes, both PI3 K/AKT/mTOR and JNK/ERK/P38 pathways undergo changes. Drugs targeting these pathways, such as Danshen and Sanqi, can function as antioxidants by modulating these signaling pathways [68]. The DJ-1 molecule is often studied for its role in preventing oxidative stress in pericytes. DJ-1 can protect retinal pericytes from high glucose-induced oxidative stress through the Nrf2 signaling pathway [69]. PARK7, the encoding molecule for DJ-1, works synergistically to promote pyruvate dehydrogenase activity [70], inhibit oxidative stress, and prevent pericyte apoptosis via the PI3K/AKT/mTOR pathway [71]. Thus, developing drugs targeting this molecule could likely produce effective antioxidants for pericytes.

Some molecules mediate protein modifications, thereby altering the damage caused by oxidative stress. For example, DNMT1 mediates the methylation of PPARα, which exacerbates retinal damage. Drugs targeting DNMT1 or reducing PPARα methylation may become future candidates for alleviating oxidative stress in pericytes [72]. Uncoupling proteins (UCPs) and MnSOD, as proteins related to antioxidants, can have compensatory effects when their expression is modified, thereby reducing oxidative stress damage in pericytes. Drugs that enhance their compensatory functions may also serve as potential antioxidants [73]. Although there is less research on the mechanisms and drugs for combating oxidative stress in pericytes of the cochlear blood-labyrinth barrier, we can refer to studies on the blood–brain barrier and blood-retinal barrier. This could help develop more redox mechanisms for the blood-labyrinth barrier, leading to the discovery of better antioxidant drugs to reduce hearing loss caused by oxidative stress.

In summary, this experiment has completely confirmed at the organismal level that with prolongation of the diabetic course, if auditory function gradually declines, the integrity of the blood labyrinth barrier structure of the cochlea will be damaged, leading to increased patency. At the cellular level, the mitochondrial membrane potential, ROS content, cleaved caspase-3 and Bax protein expression in the cochlear pericytes gradually increased with the prolongation of high glucose intervention time, and CytC and AIF in the mitochondria were continuously released to the cytoplasm. After the treatment with NAC, the above conditions were alleviated. These results suggest that high glucose induces pericytes apoptosis by increasing mitochondrial ROS content and activating both caspase-dependent and non-dependent mitochondrial apoptosis pathways, thus affecting the permeability of the blood labyrinth barrier, providing experimental evidence for the possible mechanism of diabetic hearing loss.

## Ethics statement

This study was approved by the Medical Ethics Committee of First Affiliated Hospital, School of Medicine, Shihezi University (A2022-167-01).

## Author contributions

T.F.S., and Z.Z. designed the study. T.F.S. wrote the paper. Z.Z. prepared the figs. L.L. and J.Q.S. revised the article. T.F.S., Z.Z. and W.J.J. performed the experiment. Z.Z. and T.L.H. provided materials and reagents for the study. All authors have read and agreed to the published version of the manuscript.

## Supplementary Material

Supplemental Material

## Data Availability

The data that support the findings of this study are available from the corresponding author upon reasonable request.
