# Supplementary material for: Hyperglycemia-induced oxidative stress exacerbates mitochondrial apoptosis damage to cochlear stria vascularis pericytes via the ROS-mediated Bcl-2/CytC/AIF pathway
Source: Redox Rep. 2024 Aug 2;29(1):2382943. doi: 10.1080/13510002.2024.2382943 (PMC11299461; doi:10.1080/13510002.2024.2382943)
Supplement: Supplemental Material [file YRER_A_2382943_SM9018.docx]

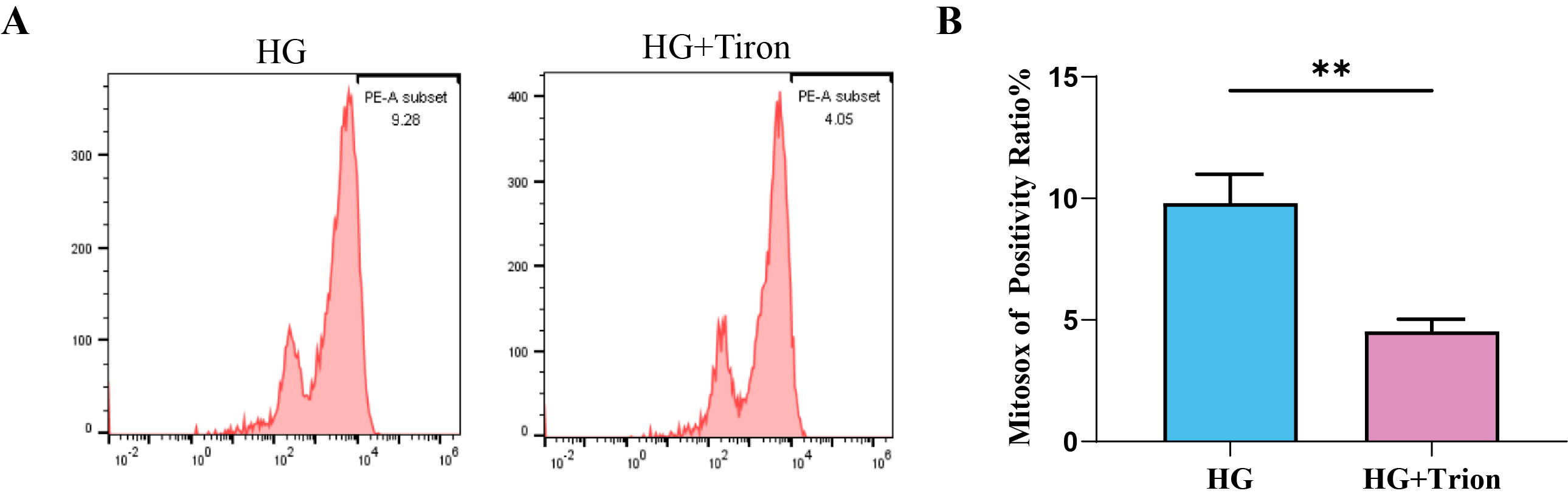


**Supplementary Figure S1.** MitoSox can monitor mitochondrial ROS. A:Representative flow cytometry image combining Mitosox; B: Statistical analysis of mitochondrial ROS content, n =3, two-tailed unpaired student's t-test, ^**^P < 0.01. Data are presented as the means ± SEMs.
